# Tumor suppressor genes are frequently methylated in lymph node metastases of breast cancers

**DOI:** 10.1186/1471-2407-10-378

**Published:** 2010-07-20

**Authors:** Weiwei Feng, Rosaria Orlandi, Naiqing Zhao, Maria Luisa Carcangiu, Elda Tagliabue, Jia Xu, Robert C Bast, Yinhua Yu

**Affiliations:** 1 Obstetrics and Gynecology Hospital of Fudan University, Shanghai, China; 2 Fondazione IRCCS Istituto Nazionale dei Tumori, Milan, Italy; 3 Department of Bioinfomatics, Shanghai Medical College, Fudan University, Shanghai, China; 4 Department of Experimental Therapeutics, The University of Texas, M.D. Anderson Cancer Center, Houston, Texas, USA

## Abstract

**Introduction:**

Metastasis represents a major adverse step in the progression of breast carcinoma. Lymph node invasion is the most relevant prognostic factor; however little is known on the molecular events associated with lymph node metastasis process. This study is to investigate the status and role of methylation in lymph node metastatic tumors.

**Materials and methods:**

Bisulfite pyrosequencing is used to screen 6 putative tumor suppressor genes (*HIN-1, RASSF1A, RIL, CDH13, RARβ2 *and E-cadherin) in 38 pairs of primary breast tumors and lymph node metastases.

**Results:**

We found that *HIN-1, CDH13, RIL, RASSF1A *and *RARβ2 *were frequently methylated both in primary and metastatic tissues (range: 55.3%~89.5%). E-cadherin was not frequently methylated in either setting (range: 18.4%~23.7%). The methylation status of *HIN-1, CDH13, RIL*, and *RARβ2 *in lymph nodes metastasis were correlated with that in primary tumors. The Pearson correlation values ranged from 0.624 to 0.472 (*p *values < 0.01 to 0.001). Interestingly, we observed an association between *HIN-1 *methylation and hormone status in metastatic lymph nodes. Hypermethylation of *HIN-1 *in metastasis lymph nodes was significantly associated with expression of ER (odds ratio, 1.070; P = 0.024) and with PR (odds ratio, 1.046; P = 0.026).

**Conclusions:**

This study suggests that hypermethylation of tumor suppressor genes is extended from primary to metastatic tumors during tumor progression.

## Background

Breast carcinoma is the most common malignancy among women worldwide. Metastasis represents an important step in the progression of fatal disease [1]. Metastases are formed by cancer cells from the primary tumor mass that travel through blood and lymphatic vessels to colonize lymph nodes, bone, lung, liver and brain. Complex genetic and epigenetic alterations affect the efficiency of each step in tumor progression. Clinical detection of distant metastasis is uncommon at presentation, but regional lymph node metastases are detected more frequently and correlate with the risk of subsequent recurrence at distant sites. Molecular analysis of metastatic lesions is gradually increasing our understanding of the events underlying the distant spread of breast cancer cells from primary cancers.

Genetic changes that occur in metastatic cells have been studied at the level of individual genes, tissue specific profiles and whole genome approaches [2]. In general, metastases and primary cancers have exhibited very similar expression signatures. The resemblance between primary and secondly metastasis lymph nodes provide evidence that the fundamental biological processes which shape the emergence of the metastatic phenotype have some underlying homologies. But some reports revealed a small number of genes that are differentially expressed between primary and metastasis even there were discrepancies in different studies, indicating potential mechanistic importance during metastasis event [2-5].

By contrast, epigenetic alterations in metastases are less characterized than genetic changes in primary cancers. In the last decade, multi gene methylation in breast primary tumors has been well-documented [6], but only small sets of genes have been shown to be methylated both in the primary tumor and in breast cancer metastasis. For example, down-regulation of tumor suppressor gene *FEZ1/LZTS1*involves promoter methylation and has been found in lymph node metastases [7]. Epigenetic silencing of *DFNA5*, encoding chemokine *CXCL12*, may contribute to the metastatic progression of breast carcinomas [8-10]. A higher prevalence of E-Cadherin, *RASSF1A, RAR-β2, APC, TWIST *and *GSTP1 *methylation in primary cancers has been associated with sentinel lymph node metastasis [11].

There are, however, only few studies that compare methylation profiles of metastases with those of the matched primary breast cancer. Metge et al. reported that 45% of the primary tumors and 60% of the matched lymph node metastases displayed hypermethylation of the *BRMS1 *promoter [12]. Mehrotra found that lymph node metastasis had a trend of high prevalence of methylation compared to the primary breast carcinoma [13]. In addition, epigenetic silencing of *CST6 *is more frequently observed in metastatic lesions than in primary cancers [14]. Furthermore, Rodenhiser et al [9] generated more intense methylation signatures related in lymph node metastasis using a highly metastatic variant (MDA-MB-468GFP-LN; 468LN) of a poorly metastatic MDA-MB-468GFP human breast adenocarcinoma cell line [15]. Most studies have used non-quantitative assays of methylation that can provide only the prevalence of methylation in primary and metastatic lesions. A quantitative comparison of methylation levels for specific genes in primary and metastatic cancers has generally not been performed.

Our previous study demonstrated that 5 putative tumor suppressor genes (*HIN-1, RASSF1A, RIL, CDH13, RARβ2*) were frequently methylated in primary breast cancers. We also identified two patterns of methylation that correlated either positively (*HIN-1/RASSF1A*) or negatively (*RIL/CDH13*) with ER/PR status [16]. We hypothesized that if silencing of these suppressor genes were important for tumor progression, hypermethylation of their promoters would be observed in lymph node metastasis at least as often and possibly more frequently than in the primary tumor. To test this hypothesis, we used bisulfite pyrosequencing to determine methylation of the 5 putative tumor suppressor genes as well as another metastasis-related gene E-cadherin in 38 pairs of primary breast tumors and lymph node metastases. We found that five of the 6 genes were frequently methylated at both sites and similar levels of methylation were found in lymph node metastases and in the primary cancers.

## Methods

### Tissue samples

Matched primary breast cancers and their lymph node metastases were obtained from 38 patients who underwent surgery between 1989 and 2007 at the Fondazione IRCCS Istituto Nazionale dei Tumori (Milan, Italy). Thirty four normal breast epithelial controls were provided by the Breast Cancer Tumor Bank at U.T.M.D. Anderson Cancer Center. These normal breast tissues were from breast cancer patients who had undergone surgery in 2004 to 2005, all normal samples were collected at least 3 cm away from the site at which the tumor was sampled and confirmed its normality by two pathologists. All tissue samples were obtained after receiving informed consent, according to institutional rules. The study was approved by the Medical Ethics Committee of the Fondazione IRCCS Istituto Nazionale dei Tumori (Milan, Italy) and the Institutional Review Board of the University of Texas, M.D. Anderson Cancer Center. Samples were grossly dissected, frozen within 1 hour of surgery, and stored at -80°C.

Studies on matched primary tumors and their metastastic lymph nodes from the same patients are rare and severely limited in the number of samples because of the difficulties of obtaining suitable specimens. The 38 breast cancer samples with their paired lymph node metastases of the present study were selected from over 1500 specimens from human breast cancer patients by careful screening of clinical criteria and histopathological features. The criteria of selection of paired primary tumor and lymph nodes were the following: lymph nodes with enough tumor tissue for both diagnosis and research, presence of a single primary tumor and lymph nodes removed in the same surgery or in a following surgery no more than 2 month later. The lymph node samples often contained only benign tissues, massive infiltration of inflammatory cells or necrotic tumors. Some metastasis lymph nodes contained small percentage of malignant tumor cells. Thus, less than 50% of samples collected were considered of quality suitable for molecular analysis.

The chosen samples were confirmed to contain sufficient tumor tissue for methylation analysis and to be free of necrotic and fibrous material. The first 10 μm frozen section from the block face was stained with hematoxylin and eosin for pathological assessment. Two pathologists examined each section and independently assessed the percentage area occupied by the malignant cells. The results were pooled for each piece of tissue and averaged to obtain final values. The lymph nodes of 2 cases (case 19 and case 21) were confirmed to contain only inflammatory cells. Thus these 2 cases were excluded and left 38 pairs of matched primary tumors and metastasis lymph nodes. The fraction of cancer cells in pairs of primary and metastastic lymph nodes were comparable, ranging from 60%-80%. As sample availability was highly limiting, the collected cohort represents a variety of tumor types, pathological characteristics and clinical treatments.

### Immunohistochemical analysis

ER and PR status was determined on primary tumors by immunoperoxidase staining using ER-ICA/PGR-ICA assay (Abbott, Abbott Park, Illinois) according to the manufacturer's recommendations. Tumors were considered receptor-positive if more than 10% of malignant cells showed nuclear staining.

HER-2/neu status was determined on both primary breast carcinomas and metastastic lymph nodes specimens by immunohistochemistry using A0485 Polyclonal rabbit anti-Human c-erbB-2 antibody (Dako, Denmark). Immunoperoxidase assays were carried out by a sensitive peroxidase-streptavidin method on formalin-fixed, paraffin-embedded sections of breast carcinomas. Briefly, 1-2 μm consecutive sections were cut, deparaffinized, rehydrated, and pretreated using the heat-induced epitope retrieval method. Endogenous peroxidase activity was blocked by 0.3% hydrogen peroxide in methanol for 30 min. After several washes in PBS and treatment with normal goat serum (1:50) for 30 min at room temperature, sections were incubated overnight at 4°C with primary monoclonal antibody, followed by biotinylated anti-mouse IgG and streptavidin conjugated horseradish peroxidase (Dako, Denmark). Peroxidase activity was detected using 3,3'-diaminobenzidine as substrate. Negative controls were incubated without primary antibody. Staining was quantified using a score of 0, 1, 2, 3 and 3+ tumors were considered as HER2 positive tumors.

### DNA extraction and sodium bisulfite treatment

Genomic DNA was extracted from 50 mg portions of each frozen tissue sample of primary and metastatic tissues using a Dneasy tissue kit (QIAGEN, Valencia, CA) and quantitated spectrophotometerically. Bisulfite treatment of 1-2 μg of genomic DNA was performed as previously described [17].

### Genes studied and Pyrosequencing methylation analysis

Five tumor suppressor genes: *RASSF1A, HIN-1 (SCGB3A1), RARβ2*, *CDH13*, *RIL (PDLIM4) *were selected for this study, as they are frequently methylated in primary breast carcinomas [16]. Another metastasis-related gene E-cadherin (*CDH1) *also included in this study.

Global methylation was estimated by testing methylation level of *LINE1 *repetitive elements. Bisulfite pyrosequencing was used to detect methylation of all 7 genes. The primers for pyrosequencing and PCR conditions are descried previously [13]. Two to six CpG sites were studied for each particular CpG island. Bisulfite-treated DNA (1 μl) was amplified in 50 μl of reaction mixture, containing primers and 0.2 units of Tag polymerase (New England Biolabs, Ipswich, MA). For the amplification of *HIN-1, RARβ2, CDH1*, we used a universal primer approach [18].

The PCR product was purified and methylation was quantitated using the PSQ HS 96A pyrosequencing system and Pyro gold reagents (Biotage, Westborough, MA). Methylation data are presented as the percentage of average methylation in all observed CpG sites. To set the controls for pyrosequencing, we used cancer cell lines and normal cells that were consistently positive or negative with stable levels of methylation. In this study, each PCR assay included a positive control (the MDA-MB-231 breast cancer cell line, which is highly methylated in most genes) and a negative control (normal breast epithelial cells, HMEC231, which are unmethylated in each of the six genes, but methylated in *LINE1). *Accuracy, reproducibility and quantitation of bisulfite pyrosequencing results were evaluated as described [16].

### Analysis of expression data from Ellsworth dataset

Expression data of *RASSF1A, RARβ2*, E-cadherin *(CDH1), CDH13*, *RIL *(*PDLIM4) *genes were extracted from Ellsworth's dataset [3] containing microarray data from microdissected tumor cells of 20 primary tumors and their corresponding lymph node metastasis analyzed on Affimetrix HG U133A 2.0 arrays. *HIN-1 *was absent in the dataset. *RIL, RARβ2 *and E-Cadherin, genes were recognized by multiple probesets. Two-sample t test was used to compare value of gene expression in primary tumors and lymph node metastasis.

### Statistical methods and analysis

Descriptive analyses were performed first for exploratory purposes. Binomial exact test was used for paired comparison. Spearman's correlation coefficient was used to assess the relationship between two continues variables. Pearson Chi-square tests or Fisher tests were used to assess the association between two categorical variables. Two-sample t test was applied to compare the means of gene methylation between tumor characteristics. Multivariate logistic regression was performed to assess whether the various levels of gene methylation were associated with the ER, PR, HR, or HER-2/neu status.

We used Cox model for survival analysis to assess whether the various levels of gene methylation were associated with disease free survival and overall survival. All reported *p *values are two-sided and considered statistically significant if *p *< 0.05. Analyses were performed using Stata 8.0 software.

## Results

### Clinical and pathological characteristics of breast cancer cohort of patients

Thirty-eight cases of primary tumor and the respective metastatic axillary lymph node were selected using the criteria listed in Materials and Methods. The clinicopathologic features of 38 cases are presented in Table 1 and Additional file 1 Table S1. The average age at diagnosis was 59 years (ranged 35-84 years), which was comparable with that of normal controls (average age was 60 years, ranged 29-80 years). Twenty eight cases were ductal carcinomas, 4 were lobular carcinomas, 4 were mixed and 2 were apocrine and neuroendocrine cancers. The ER/PR status was assessed in all primary tumors: 79% of cases were ER positive, whereas 60% were PR positive. HER-2/neu was overexpressed in 26% of primary tumors and 33% in metastatic lymph nodes. Thirty-four patients were followed up from 1 year to 10 years, whereas 4 cases were lost.

**Table 1:** Summary of clinical and pathological characteristics of 38 breast cancer patients

Clinicopathologic factors	Number of sample
Age	
Mean	59 years
range	35-84 years
Histology	
Ductal	28
Lobular	4
Mixed	4
Other	2
Clinical stage	
I	0
II	12
III	17
IV	9
Tumor size	
< 2 cm	11
> 2 cm	27
Grade	
I	0
II	16
III	22
ER Status	
Positive	30
Negative	8
PR status	
Positive	23
Negative	15
HER-2/neu primary tumor	
Positive	10
Negative	28
HER-2/neu lymph node metastasis	
Positive	11
Negative	22
N/A	5
Follow up status	
Progression (recurrence, metastasis, death)	14
No evidence of disease	20
Lost to follow-up	4

N/A: not tested

**Figure 1:**
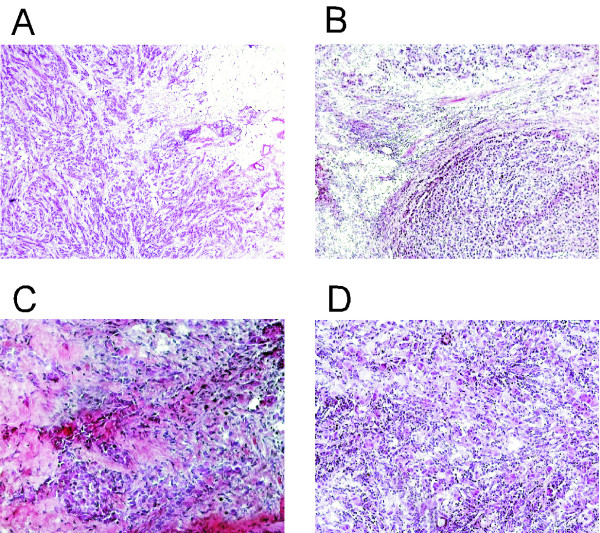
**Pathologic morphology of two frozen primary breast carcinoma (A, C) and their corresponding metastatic axillary lymph node (B, D) by hematoxylin and eosin staining**.

Two pathologists reviewed all samples independently; the proportion of malignant cells in each pair of samples was comparable, with 34 pairs showing more than 80% malignant cells. The representative photographs of frozen sections of two primary tumors and their matching lymph node metastasis confirmed the high quality of samples (Figure 1)

#### Methylation of putative suppressor genes

Using pyrosequencing, we measured the methylation status of 6 putative tumor-suppressor genes *HIN-1*, *CDH13*, *RIL, RASSF1A*, *RAR*β*2*, and E-cadherin. Overall, 38 pairs of primary and metastatic breast cancers were assayed. The sample mean plus two times the standard deviation of the pooled normal samples was used to choose the following cutoffs: *HIN-1 *(10.9), *CDH13 *(10), *RIL *(16.9), *RASSF1A *(10), *RARβ2 *(10), E-cadherin (10). Table 2 showed that *HIN-1, CDH13, RIL, RASSF1A *and *RARβ2 *were frequently methylated both in primary and metastatic tissues (range: 55.3%~89.5%). E-cadherin was not frequently methylated both in primary and metastatic tissues (range: 18.4%~23.7%). A similar fraction of methylated genes was observed in the primary cancers and lymph node metastases, no difference between these two groups of each gene.

**Table 2:** Methylation positive rates of 6 tumor suppressor genes in primary cancer tissues and lymph nodes metastasis

Genes	Number of methylated cases (positive rate %)		
	
	Primary cancer	Lymph nodes metastasis	*p *value*
*HIN-1*	28 (73.7)	26 (68.4)	0.508

*CDH13*	32 (84.2)	34 (89.5)	0.688

*RIL*	25 (65.8)	25 (65.8)	1.000

*RASSF1A*	25 (65.8)	24 (63.2)	1.000

*RARβ2*	23 (60.5)	21 (55.3)	0.774

E-cadherin	7 (18.4)	9 (23.7)	0.754

*Binomial exact test for paired comparison.

#### Correlation of methylation in the primary breast cancers and lymph node metastases

**Figure 2:**
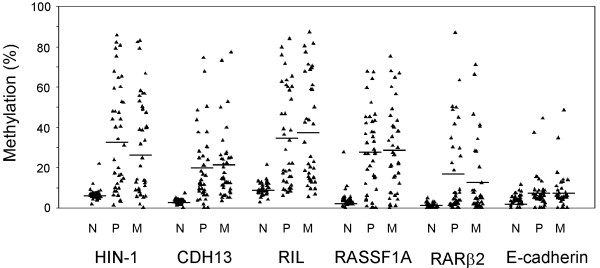
**Comparison of methylation levels of 6 genes in normal breast (N), paired primary tumors (P) and metastatic lymph nodes (M)**. Mean methylation level of N, P, and M of each group was provided by horizontal lines.

To study whether gene methylation in lymph node metastases correlated with that in primary cancers, Pearson's correlation coefficient was used to assess the relationship between two continuous variables. Table 3 displays mean methylation levels for each gene and the correlation between the two sites. Among these genes, the methylation status of *HIN-1, CDH13, RIL *and *RARβ2 *in lymph nodes metastasis was correlated with that in primary tumors. The Pearson correlation values ranged from 0.624 to 0.472 (*p *values < 0.01 to 0.001). *LINE-1*, as a global methylation marker, also correlated in primary and metastatic tumors. Figure 2 shows the methylation levels in normal, primary tumor and lymph nodes metastasis. A statistically significant correlation was not observed with *RASSF1A *or with E-cadherin.

**Table 3:** Methylation levels of 7 genes in primary breast cancer tissues and lymph nodes metastasis

Genes	Normal	Methylation level (mean ± SD)		Pearson correlation coefficient	*p *value
				
		Primary cancer	Lymph nodes metastasis		
*HIN-1*	6.5 ± 3.2	36.4 ± 26.5	33.1 ± 24.2	0.624	< 0.001

*CDH13*	3.0 ± 1.2	20.0 ± 17.9	20.6 ± 17.5	0.545	< 0.001

*RIL*	9.1 ± 3.4	34.5 ± 24.6	36.8 ± 26.2	0.547	< 0.001

*RASSF1A*	4.0 ± 4.7	28.0 ± 19.8	28.3 ± 21.3	0.233	0.16

*RARβ2*	1.1 ± 1.2	16.4 ± 21.6	12.3 ± 18.5	0.472	0.003

E-cadherin	4.0 ± 2.5	7.8 ± 8.8	7.8 ± 9.1	0.093	0.583

*LINE-1*	66.8 ± 3.4	58.3 ± 9.8	58.3 ± 10.9	0.614	< 0.001

**Figure 3:**
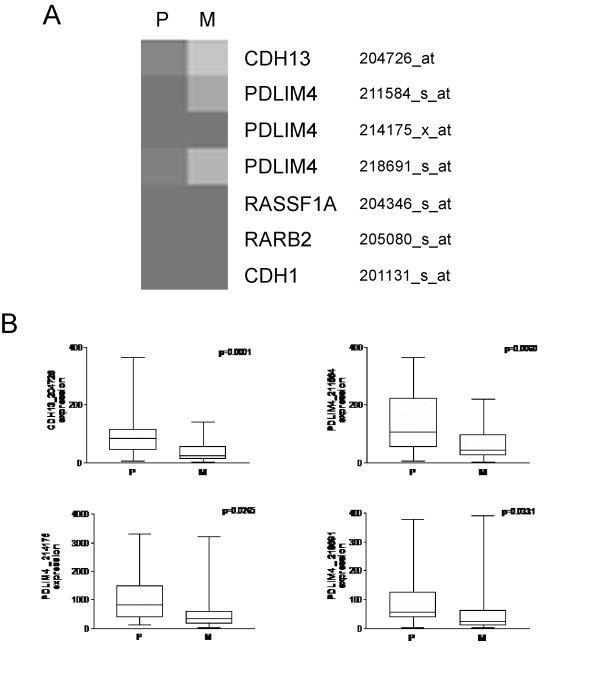
**Expression of *CDH13, RIL (PDLIM4), RASSF1A, RARβ2 *and E-cadherin *(CDH1) *in primary (P) and matched lymph node metastasis (M) samples from Ellsworth's study **[3]. (A) Heat map of expression levels of the 5 genes analyzed. Gene symbols and probesets are reported. (B) Box-plot of expression of *CDH13 *and *RIL (PDLIM4) *genes in primary tumors and lymph node metastasis. *PDLIM4 *gene was recognized by multiple probe sets. P = primary; M = lymph node metastasis.

To evaluate the expression of 6 genes in primary and metastatic breast tissues, expression values of those genes were searched in the dataset originated by the microarray analysis of Ellsworth et al. [3] on laser microdissected tumor cells from 20 primary and their corresponding lymph node metastasis. *HIN-1 *was absent in the dataset. *RASSF1A, RARβ2*, E-cadherin *(CDH1), CDH13 *and *RIL *were found to be expressed at a low level, in agreement with the hypermethylation of these genes in tumor tissues. Expression values of selected genes ranged from 1.1 to 3302.50 (mean 164.59, SD 372.65), whereas the global gene expression range was 0-47165.60 (mean 708.25, SD 2044.49). The expression of *CDH13, RIL (PDLIM4), RASSF1A*, RARβ2 and E-cadherin *(CDH1) *in primary tumor and their lymph node metastasis is compared in Figure 3. *CDH13 *and *RIL (PDLIM4) *expression levels were significantly decreased in lymph node metastasis, whereas no significantly changes in the expression of *RASSF1A, RARβ2 *and *CDH1 *mRNAs were observed.

#### Correlation of methylation between different genes

On the basis of data from continuous marker methylation analyses in primary breast cancer tissues, the methylation levels of *RASSF1A *and *HIN-1 *exhibited a strong correlation to each other (R = 0.502, *p *< 0.01). Some moderate correlations were found between *RASSF1A *and *CDH13 *(*R *= 0.317, *p *< 0.01) and between *RASSF1A *and *RIL *(R = 0.497, *p < 0.01*). Similarly, in lymph nodes metastasis, a moderate correlation existed between *RASSF1A *and *RIL *(R = 0.489, *P <*0.01), *CDH13 and *E-cadherin (R = 0.391, *p <*0.01); *RASSF1A *and *CDH13 *(R = 0.315, *p <*0.01).

### Relationship between hormone receptor status and methylation in primary breast cancers and lymph nodes metastasis

Our pervious study reported that in the primary breast tumors, the methylation of the *HIN-1/RASSFIA *panel strongly correlated positively with the expression of ER, PR, and HR. Conversely, the methylation of the *RIL/CDH13 *panel correlated negatively ER, PR, and HR expression [16]. In this study, we investigated if this association exists in metastatic lymph nodes. By t-Test analysis, it is showed that patients with a positive ER status had higher methylation levels of *HIN*-1 in primary tumors (mean of 41.3 in ER-positive cases versus 15.8 in ER-negative cases, *p *= 0.001). Higher methylation levels of *RIL *in primary tumors were also found in PR negative cases (mean of 41.5) when compared with PR positive cases (mean of 30.5), but no statistical difference was found, possibly related to the small sample size (Table 4).

**Table 4:** Association of hormone receptors with gene methylation in primary cancers (P)

	ER (+)	ER (-)	*p *value	PR (+)	PR (-)	*p *value
*HIN-1 *(P)	41.3 ± 26.1	15.8 ± 13.2	0.001	40.3 ± 25.0	30.1 ± 27.3	0.232

*CDH13 *(P)	18.8 ± 18.8	21.0 ± 14.0	0.756	21.0 ± 19.4	16.5 ± 15.2	0.440

*RIL *(P)	33.2 ± 24.9	41.9 ± 24.1	0.380	30.5 ± 25.3	41.5 ± 23.0	0.171

*RASSF1A *(P)	29.5 ± 20.5	21.9 ± 17.6	0.340	28.6 ± 20.0	27.0 ± 20.5	0.809

*RARβ2 *(P)	18.2 ± 23.4	11.3 ± 11.6	0.249	17.9 ± 21.2	15.2 ± 22.9	0.706

E-cadherin (P)	7.3 ± 6.6	10.5 ± 15.4	0.602	6.2 ± 4.2	10.4 ± 12.7	0.230

Interestingly, we observed an association between hormone status and gene methylation in metastatic lymph nodes. Patients with a positive ER and PR status had higher methylation levels of *HIN-1 *(mean of 35.8 in ER-positive tumors versus 15.3 in ER-negative tumors, *p *= 0.008; mean of 38.1 in PR-positive tumors versus 22.5 in PR-negative tumors, *p *= 0.047) (Table 5).

**Table 5:** Association of hormone receptors with gene methylation in lymph nodes metastasis (M)

	ER (+)	ER (-)	*p *value	PR (+)	PR (-)	*p *value
*HIN-1 *(M)	35.8 ± 24.3	15.3 ± 13.9	0.008	38.1 ± 24.7	22.5 ± 19.9	0.047

*CDH13 *(M)	17.8 ± 14.8	29.4 ± 23.8	0.220	18.8 ± 16.5	22.1 ± 18.6	0.565

*RIL *(M)	35.0 ± 24.2	42.4 ± 31.6	0.474	31.8 ± 25.3	43.6 ± 25.1	0.155

*RASSF1A *(M)	24.8 ± 20.0	35.7 ± 26.3	0.203	25.5 ± 21.4	29.2 ± 22.1	0.594

*RARβ2 *(M)	10.2 ± 13.7	18.7 ± 30.7	0.470	11.6 ± 14.7	12.5 ± 22.9	0.881

E-cadherin (M)	8.1 ± 8.6	8.2 ± 11.5	0.975	8.7 ± 9.3	7.1 ± 8.9	0.599

Using multivariable logistic regression analysis, hypermethylation of *HIN-1 *in primary tumor was significantly associated with ER positivity (odds ratio, 1.057; *p *= 0.027). Hypermethylation of *HIN-1 *in metastasis lymph nodes was also significantly associated with ER positivivity (odds ratio, 1.070; P = 0.024). Additionally, hypermethylation of *HIN-1 *in metastasis lymph nodes was significantly associated with PR positivity compared with PR negative patients (odds ratio, 1.046; P = 0.026). Hypermethylation of *RIL *in lymph nodes was negatively associated with PR positivity (odds ratio, 0.964; P = 0.044).

Using multivariable logistic regression models, we have assessed the correlation of HER-2/neu expression with methylation of each gene (*RIL*, *HIN-1*, *RASFF1A*, *CDH13*, E-cadherin and *RARβ2*). No correlation between gene methylation and HER-2/neu expression was found (data not shown).

#### Relationship between other clinical characteristics and methylation of individual genes in primary tumors and lymph nodes metastasis

Among all the genes, *CDH13 *and E-cadherin methylation in lymph nodes were positively associated with age (R = 0.467, 0.435 respectively, *p *< 0.01). Using the Logistic regression model, no statistically significant associations were found for methylation status with clinical stage, tumor size, and grade or tumor progression.

#### Relationship between survival and methylation status in primary tumors and lymph nodes metastasis

No significant association was found between methylation status and overall or disease-free survival.

## Discussion

Hypermethylation and silencing of specific putative tumor suppressor genes is clearly observed in a fraction of human breast cancers, possibly contributing to uncontrolled growth. Inhibition of DNA methylation can activate silenced tumor suppressor genes, associated with the arrest of tumor growth. Our previous study revealed that five of 12 tumor suppressor genes, *RIL, HIN-1, RASSF1A, CDH13*, and *RARβ2*, were frequently methylated in primary breast cancers but not in normal breast tissue. To determine whether hypermethylation is also found in lymph node metastasis of breast cancers, 38 pairs of primary breast tumors and lymph node metastases were selected. In these pairs, the percentage of malignant cells in primary and metastatic samples was comparable. Quantitative pyrosequencing was used to examine the methylation status in these tumors. Our results indicate that *RIL, HIN-1, RASSF1A, CDH13*, and *RARβ2 *are also frequently methylated both in primary breast cancers and in lymph node metastases. The methylation status of *HIN-1, CDH13, RIL, RARβ2 *in lymph nodes metastasis were statistically correlated with that in primary tumors (*p *values < 0.01 to 0.001). Consequently, metastatic cancer cells retained hypermethylation and silencing of putative tumor suppressor genes.

As a consequence of hypermethylation status of their promoters, the frequently methylated genes are expected to be underexpressed in both primary breast cancer and lymph node metastasis. Our preliminary quantitative PCR analysis on tumor and lymph node metastasis samples showed a very low expression of these genes. By combining a public dataset [3] containing microarray data from microdissected tumor cells of 20 primary tumors and their corresponding lymph node metastasis, we also observed low expression levels of RASSF1A, RARß2, CDH13, RIL in the paired samples, suggesting that hypermetylation of such genes may directly reflects their gene expression.

The hypermethylation and the preservation of a restricted expression or a further decreased expression in lymph node metastasis suggest that the inhibition of the tumor/invasion suppressor genes is indeed required for breast cancer progression. Carraway et al. reported that in the early stage cancer (lymph node negative) methylation changes can predict the recurrence [19]. In this study, we studied the methylation status in metastasis lymph nodes and found that methylation changes which already accumulated in primary cancer were continued in metastatic tumors. Continued methylation and silencing of growth inhibitory genes is consistent with the possibility that inhibitors of DNA methylation might reactivate their expression and inhibit growth of metastatic cancer. Our study suggested that DNA methylation detection in lymph nodes tumors would be a useful marker for demethylating drugs therapy.

Only a few reports describe the methylation status of metastatic breast cancer. Metge et al. examinated the *BRMS1 *promoter region in a panel of 20 patients samples and showed that 45% of the primary tumors and 60% of the matched lymph node metastases displayed hypermethylation of *BRMS1 *promoter [12]. Mehrotra J et al. reported that lymph node metastasis exhibited a somewhat higher prevalence of methylation compared with the primary breast carcinoma for five genes (*Cyclin D2, RAR-beta, Twist, RASSF1A*, and *HIN-1) *with a statistically significant increase in methylation of *HIN-1 *(*p *= 0.04). Compared with the primary breast carcinomas, bone, brain, and lung metastasis exhibited higher methylation frequencies, with *HIN-1 *and *RAR*-beta methylation being significantly higher (*p *< 0.01) in each group [13]. These reports were, however, based on non-quantitative methylation-specific PCR assays. Our study has used more quantitative techniques, therefore, our results are more reliable.

Our previous study reported that in the primary breast tumors, the methylation of *HIN-1 *and *RASSF1A *strongly correlated with the expression of ER, PR, and HR. Conversely, the methylation of the *RIL *and *CDH13 *strongly correlated with lack of ER, PR, and HR expression [16]. Interestingly, in this study, we further confirmed that this association also exists in metastatic lymph nodes. Using multivariate logistic regression analysis, hypermethylation of HIN-1 in lymph nodes metastases was significantly associated with ER positive cancers (odds ratio, 1.070; P = 0.024) and significantly associated with PR positive cancers (odds ratio, 1.046; P = 0.026), suggesting that methylation of *HIN-1 *gene may controlled by hormone receptors.

Taken together, our data suggest that hypermethylation and silencing of tumor suppressor genes is carried from primary breast cancers to lymph node metastases. Methylation and silencing of suppressor genes in the primary and metastatic tumors did not correlate with overall or disease free survival, but this may relate to the small sample size. A large cohort study may be required to test the prognostic significance of promoter methylation.

## Conclusions

We used bisulfite pyrosequencing to screen 6 putative tumor suppressor genes (*HIN-1, RASSF1A, RIL, CDH13, RARβ2 *and E-cadherin) in 38 pairs of primary breast tumors and lymph node metastases. Our data indicated that *HIN-1, CDH13, RIL, RASSF1A *and *RARβ2 *were frequently methylated both in primary and metastatic tissues. The methylation status of *HIN-1, CDH13, RIL*, and *RARβ2 *in lymph nodes metastasis was correlated with that in primary tumors. This study suggests that hypermethylation is extended from primary to metastatic tumors during tumor progression.

## Competing interests

The authors declare that they have no competing interests.

## Authors' contributions

WF carried out the methylation assays and prepared the manuscript; JX carried out partial methylation assays; RO provided DNA, clinical data and helped prepare the manuscript; NZ performed the statistical analysis; MLC and ET provided pathologic supports for tissue sections; YY & RCB conceived the study and prepared the manuscript. All authors read and approved the final manuscript.

## Pre-publication history

The pre-publication history for this paper can be accessed here:

http://www.biomedcentral.com/1471-2407/10/378/prepub

## Supplementary Material

Additional file 1 Table S1: Details of clinical and pathological characteristics of 38 breast cancer patients Click here for file
