# Supplementary material for: Tumor suppressor genes are frequently methylated in lymph node metastases of breast cancers
Source: BMC Cancer. 2010 Jul 20;10:378. doi: 10.1186/1471-2407-10-378 (PMC2914707; doi:10.1186/1471-2407-10-378)
Supplement: Additional file 1 — Table S1: Details of clinical and pathological characteristics of 38 breast cancer patients [file 1471-2407-10-378-S1.DOC]

Additional file 1

Table S1. Details of clinical and pathological characteristics of 38 breast cancer patients

| Sample number | Age | Pathological | Grade | ER | PR | HER-2 (P) | HER-2 (M) | DFS (month) | OS (month) | Outcome |
| --- | --- | --- | --- | --- | --- | --- | --- | --- | --- | --- |
| 1 | 65 | lobular | II | + | - | 3+ | 3+ | 1 | 5 | Dead |
| 2 | 83 | apocrine | II | + | + | 2+ | 3+ | 1 | 18 | Dead |
| 3 | 73 | ductal | III | + | + | 2+ | 2+ | 1 | 37 | Dead |
| 4 | 54 | lobular | II | + | + | 0 | 0 | 176 | 176 | NED |
| 5 | 57 | mixed | II | + | + | 2+ | 0 | 124 | 124 | NED |
| 6 | 52 | ductal | III | - | - | 1+ | 1+ | 204 | 204 | NED |
| 7 | 44 | ductal | III | + | + | 1+ | 0 | 98 | 98 | NED |
| 8 | 60 | ductal | III | + | + | 1+ | 1+ | N/A | 78 | Dead |
| 9 | 53 | mixed | II | + | + | 0 | 0 | 160 | 160 | NED |
| 10 | 60 | ductal | III | - | - | 1+ | 1+ | 60 | 82 | Dead |
| 11 | 62 | ductal | III | + | + | 1+ | 0 | 16 | 40 | Dead |
| 12 | 36 | ductal | II | + | - | 0 | 0 | 18 | 48 | Dead |
| 13 | 42 | ductal | III | + | - | 3+ | 3+ | 107 | 107 | NED |
| 14 | 46 | lobular | II | + | + | 0 | 0 | 24 | 83 | Dead |
| 15 | 64 | ductal | III | + | - | 0 | 0 | N/A | 129 | Dead |
| 16 | 54 | mixed | II | + | + | 1+ | N/A | 12 | 12 | NED |
| 17 | 36 | mixed | II | + | + | 3+ | 3+ | 11 | 44 | Dead |
| 18 | 64 | ductal | III | - | - | 0 | 0 | 132 | 132 | NED |
| 20 | 44 | ductal | II | + | + | 0 | 0 | 27 | 163 | ED |
| 22 | 66 | ductal | II | + | - | 3+ | 3+ | 110 | 110 | NED |
| 23 | 45 | lobular | II | + | + | 1+ | N/A | N/A | N/A | Lost |
| 24 | 44 | ductal | III | + | + | 0 | N/A | N/A | N/A | Lost |
| 25 | 79 | ductal | III | + | + | 2+ | 2+ | 15 | 15 | NED |
| 26 | 86 | ductal | II | + | + | 2+ | N/A | 19 | 19 | NED |
| 27 | 81 | ductal | III | - | - | 3+ | 3+ | 12 | 12 | Dead |
| 28 | 63 | ductal | III | - | - | 3+ | 3+ | 60 | 60 | NED |
| 29 | 46 | ductal | III | - | - | 0 | N/A | 52 | 52 | NED |
| 30 | 66 | ductal | III | + | + | 3+ | 3+ | 38 | 38 | NED |
| 31 | 49 | ductal | III | + | + | 2+ | 2+ | 23 | 23 | NED |
| 32 | 57 | Neuro-endocrine | III | - | - | 0 | 0 | 1 | 2 | Dead |
| 33 | 56 | ductal | III | - | - | 3+ | 3+ | N/A | N/A | Lost |
| 34 | 84 | ductal | II | + | + | 2+ | 2+ | 19 | 19 | NED |
| 35 | 61 | ductal | III | + | + | 0 | 0 | 19 | 19 | NED |
| 36 | 61 | ductal | II | + | + | 1+ | 0 | N/A | N/A | Lost |
| 37 | 64 | ductal | III | + | + | 0 | 0 | 13 | 13 | NED |
| 38 | 53 | ductal | III | + | - | 3+ | 3+ | 7 | 12 | Dead |
| 39 | 82 | ductal | III | + | - | 0 | 0 | 17 | 17 | NED |
| 40 | 69 | ductal | II | + | + | 3+ | 3+ | 13 | 13 | NED |

N/A: not tested; NED: no evidence of disease; ED: evidence of disease DFS: disease free survival; OS: total survival. P: primary tumor; M: metastasis lymph node;
